# Supplementary material for: Is the Qi experience related to the flow experience? Practicing qigong in urban green spaces
Source: PLoS One. 2021 Jan 4;16(1):e0240180. doi: 10.1371/journal.pone.0240180 (PMC7781670; doi:10.1371/journal.pone.0240180)
Supplement: Attachment — Submitted filename: Response to Reviewers.docx [file pone.0240180.s002.docx]

**Editor’s Comments:**

(--1--) Methodology of Evaluating Tests and Questionnaires

In general, all four reviewers pointed to the need of a more systematic evaluation of construct validity regarding the concepts of Qi and Flow. I would recommend presenting data and results in a more systematic fashion, using the multi-trait-multi-method (MTMM) matrix, which is a traditional approach to assessing the construct validity of a set of variables/measures related to theoretical concepts. Campbell and Fiske (1959) provided a practical methodology to be used for purposes of construct validation and the development of tests and questionnaires. At its core are two types of validity termed convergent and discriminant, which can be viewed as subcategories of construct validity. Convergent validity refers to the degree to which concepts that should be related in theory are actually interrelated through empirical observation. Discriminant validity refers to the degree to which concepts that not related in theory are actually not interrelated empirically. In order to determine construct validity of your target measure, you would have to determine both convergence and discrimination. Key to the evaluation of construct validity, however, is the analysis of test reliability as reliability coefficients are a baseline against which to interpret cross-correlations. In some way, reliability (in terms of accuracy of measurement) can be interpreted as a prerequisite to validly measure relationships with other constructs.

**RESPONSE:** We would like to express our sincere gratitude to you and to the reviewers. Your insightful suggestions and explanation of the multi-trait-multi-method (MTMM) matrix really helped us to deeply reconsider the concepts of Qi and flow. We read the suggested literature [Campbell DT, Fiske DW. Convergent and discriminant validation by the multitrait-multimethod matrix. Psychological Bulletin 1959;56(2):81-105. doi:10.1037/h0046016; Steinborn MB, Langner R, Flehmig HC, Huestegge L. Methodology of performance scoring in the d2 sustained-attention test: Cumulative-reliability functions and practical guidelines. Psychological Assessment 2008;30(3):339-57. doi:10.1037/pas0000482] and checked the matrix, step by step, finding high convergent validity but also high discriminant validity. However, in this paper, we did not intend to discriminate between Qi and flow. Our purpose was to test the correlation between Qi and flow; therefore, concerning construct validity, we not only used Table 1 to systematically evaluate the definitions and meanings of traits in Qi and flow but also invited Qigong masters and researchers to assess the construct and content validity. We then used statistical methods to verify reliability in both questionnaires. We added these statements in the revised version in the first paragraph of “Discussion” to provide the scope and explanation construct validity in the study. We hope you will be pleased with our explanations and revisions.

(--2--) Evaluation of the concept of Qi

As R2 indicates, it is important to show at the same time what Qi is and what it is not. More practically, in order to defend a proposition about what a test measures (in your case Qi), one looks basically for two things. The first is convergence of indicators. Let me illustrate this point. For example, in order to justify that a questionnaire measures Qi, the concepts is first defined analytically (through thinking about what it is) and then is set into a hypothetical space of related concepts, like for example, flow state, relaxation, or other related hypothetical states. In the next step, one has to collect data from other instruments claimed to measure these closely related constructs and to compare them by means of correlation analysis. The second point refers to discrimination. That is, scores identified with supposedly distinct states should not correlate to highly. For example, a test claimed to measure "creative state" or "alertness" should reveal some but not too high correlations with the target construct (Qi) and is therefore interpreted as to indicate discriminant validity. Another point refers to aspects of measurement accuracy. Crucially, the MTMM logic (see Campbell & Fiske, 1959) requires that measures are basically reliable, which again is dependent on the number of measurement units under the score (see Steinborn, Langner, Flehmig, & Huestegge, 2018). Low or even zero correlations cannot be interpreted as indicating discriminant validity if one or both measures lack test reliability. In other words, convergent and discriminant validity can only be interpreted by simultaneously considering reliability as the underlying basis. This means that low correlations can indicate both independence of the constructs and/or low reliability of (at least one) of the constructs. With regard to your study, I would suggests reworking the manuscript towards a more systematic step-by-step evaluation following the standard methodology of concept validation. [suggested literature: Campbell, D. T., & Fiske, D. W. (1959). Convergent and discriminant validation by the multitrait-multimethod matrix.  Psychological Bulletin, 56(2), 81-105. doi:10.1037/h0046016; Steinborn, M. B., Langner, R., Flehmig, H. C., & Huestegge, L. (2018). Methodology of performance scoring in the d2 sustained-attention test: Cumulative-reliability functions and practical guidelines. Psychological Assessment, 30(3), 339-357. doi:10.1037/pas0000482].

**RESPONSE:** We would like to thank you for your constructive comments and description of the concept of convergence and discriminant of indicators in detail. We found that using the MTMM could strengthen our discourse. Thus, in the article, we have made every attempt to fully address “what Qi is,” “what a test measures on Qi traits,” and “the similar conceptual validity of Qi and flow.” Also, in the “Methods,” the MTMM was used to construct convergent validity and discriminant validity, yielding high reliability and convergent validity; however, a slight difference was detected for discriminant validity. We explain the Qi experience as a holistic body-mind-spirit experience, one that is related to physical and mental benefits. It would be difficult to discriminant between the traits in the Qi experience questionnaires.

(--3--) Theory of Qi

An essential point concerns the distinction of the concept of Qi from a theoretical perspective. All reviewers pointed out that the concepts of Qi seems important but at the same time is in need of a more systematic theoretical evaluation. What is it that is meant by Qi relative to Flow? Also, it should be evaluated whether the correlation between Qi and Flow is due to the similarity of items under the scores of both concepts (this point was raised by R3 and R4), and if so, whether this is intended, not intended, or due to a natural conceptual overlap between the concepts. I suggest elaborating more on the theoretical nature of the concepts of Qi and Flow and delineating ways of how to measure both concepts empirically.

**RESPONSE:** Thank you for the opportunity to revise our paper once more for PLOS ONE. We totally agree with all the reviewers. The theory of Qi and its relationship to the flow experience are indeed the main concepts that we should identify more clearly in our manuscript. We revised the structure of the Introduction, added a new paragraph, lines 51–58, to compare similar meditative activities, added prior scientific research that measured Qi, in lines 80–83, and made a systematic comparison of concept validity between the flow and Qi experiences in Table 1 and lines 126–132 to explain the relationship between the concepts. We then discuss the research aims and scope in lines 136–154. In “Methods,” we added more detailed information to describe how we measured these two concepts—for example, what criteria did we use to recruit the participants? What instructions did we provide in the consent form? and also added that all data were analyzed anonymously in this study. We also added the statistical sub-scores for the Qi and flow experiences and revised the structure of the “Discussion” and “Conclusion” to more specifically discuss the results concerning the conceptual overlap between the two concepts. We appreciate all the reviewers for giving us the chance to deeply think about the concepts of Qi and flow. We believe the revised version provides better information than the last version.

**Reviewer 1 Comments:**

Reviewer #1: There are a few edits in language that should be done. I appreciate this article because, usually the concept of Qi -- important but not understood in the West -- is discounted. This article attempts to bring Qi into the dialogue. This is an excellent contribution.

**RESPONSE:** Thank you, reviewer Dr. Roger Jahnke, for your time and energy in reviewing our manuscript. We appreciate your comments and affirmation. We hope our revised and edited version is clearer for readers.

**Reviewer 2 Comments:**

Reviewer #2: Background:
The study aims to examine if the western concept of Flow is related to the eastern concept of Qi. To this end, the authors created a Qi-questionnaire. Subjects had to fill-out this questionnaire after doing Qi-Gong as well as a modified Flow-Questionnaire. The correlation between the two questionnaires shows a very high correlation showing a conceptual overlap of the two concepts. I enjoyed reading the document and was especially intrigued by the idea of similar psychological mechanisms in western and eastern concepts. Furthermore, I applaud the effort to expand the psychological toolbox by constructing new questionnaires. However, I would recommend improving the document in several aspects including literature analysis, structure, conceptual analysis, methodological description, and discussion. I can recommend this work after major revisions. Please see my comments below for further detail.

**RESPONSE:** Thank you, reviewer Jonas Ebert (M. Sc.), for providing these insights and suggestions. We agree with you and have incorporated your suggestions throughout our paper. We have clarified the concepts of Qi and flow in the “Introduction,” “Methods,” and “Conclusion,” restructured our discourse, and edited for grammar and other formulations. The following is our point-by-point response to your questions and comments. Please feel free to review our revised manuscript. We appreciate your time and effort. Thank you.

[1] Conceptual analysis
In my opinion, the paper lacked a deeper analysis of the two concepts in question. When comparing psychological mechanisms, a deep analysis and precise definitions are helpful for the reader better to understand the comparison. This is especially important when dealing with (to western audiences) unfamiliar concepts like Qi, Tao, and Qigong. For example, I was wondering about what physical activity is Qigong, especially in comparison to Thai Chi, Yoga, or similar activities.

**RESPONSE:** We agree that focusing on the analysis of the Qi and flow concepts strengthened our manuscript and allowed us to make a greater theoretical and practical contribution. We revised the first paragraph of the Qi and flow section and added a new paragraph, lines 51–58, to compare the similar meditative activities. In lines 77–89, we discuss the research scope of the study. Moreover, under the heading, “The Comparison of the Flow and Qi Experience Concepts,” we systematically compared and evaluated the construct validity of the Qi and flow experiences (lines 114–132, Table 1).

[2] Literature analysis
In addition to [1], extended literature analysis could help to clarify definitions and concepts. Especially for well-known concepts like Flow, I would expect a large amount of helpful scientific literature. I could imagine that a similar relationship like the one focused on in this paper can be found regarding other physical activities as well. It would be interesting, for example, if western sports evoke Flow or to analyze the positive benefits of Qigong, Yoga, Tai Chi, etc. Overall, I would recommend expanding the literature.

**RESPONSE:** We have made every attempt to address these comments in the revised manuscript. For instance, we added lines 71–76 to explain how urban green space plays a role in the Qi experience. In lines 80–89, we explain what the Qi experience is through its physical outcomes and use quantitative findings to point out its psychological outcomes. However, since there is limited research on meditative activities that evoke the flow experience, we present two scientific studies on yoga and Qigong that are related to the flow experience in lines 109–112. Moreover, we removed the original Table 1 and added a new Table 1 to discuss the concept in Qi and flow in lines 126–132.

[3] Structure
The reading, although a good read overall, was sometimes difficult to follow. I would recommend improving the structure of arguments for example by introducing more headlines and straighten the line of arguments. Sometimes different paragraphs would discuss the same topic or paragraphs would switch their line of argument unexpectedly to different content.

**RESPONSE:** We honestly feel that your insightful suggestions helped us to improve the structure of our manuscript. In the beginning of one paragraph (lines 30–50), we illustrate the concepts of the Qi and flow experiences and then summarize our discussion with our research scope (lines 59–66). We added the following sections to improve our arguments: “Qigong and the Essence of the Qi Experience” (line 67), which extends understanding of Qigong and the essence of the Qi experience in its physical and psychological outcomes; “The Concept of the Flow Experience” (line 90), which describes flow and related meditation research; “The Comparison of the Flow and Qi Experience Concepts” (line 113), which states in more detail the psychological mechanisms of flow, refers to a few studies on flow and meditative activities, and systematically compares the concepts of the Qi and flow experiences, as revised in Table 1; and “Aim and Scope of the Study” (line 135), which makes a clear statement from the literature review of our study. For the methods, we reorganized and added more detailed information in “Methods” about how to construct the Qi experience (starting at line 174), and we also used MTMM to assess convergent and discriminant validity (lines 187–207). For the results, we provided more details of the statistical analysis. In the discussion, we added the sections “A Possible Explanation for the Results” (line 375) and “Limitations and Recommendations for Future Research” (line 402) to clarify our results. For the conclusion, we restructured our findings and explanations.

[4] Table 1
I stumbled about table 1’s content and especially its size. It seems the content of table 1 can on the one hand be discussed in the continuous text (comparison Qi – Flow) or on the other hand, can be presented in the annex (open-ended data). Perhaps the 14 questions regarding the different aspects of Qi are the most suitable content for table 1.

**RESPONSE:** Thank you for the suggestions. The original Table 1 was separated as discussing in the continuous text (comparison Qi – Flow) in line 133 (Table 1) and the open-ended data of Develop Qi experience questionnaires (QEQ) in Table 2.

[5] Methodological concerns
I wonder how exactly the data collection as well as the correlation analysis between Qi and Flow was done, as the obtained correlations are incredibly high. Unfortunately, the description of the data collection and the statistical analysis is rather short. I suggest providing some more information with this regard. The following methodological issues should be discussed or at least reported:
1. Sequencing effects could influence the subjects’ judgments. It is however unclear in what order subjects answered the two questionnaires.
2. Expectation effects could influence judgments. However, it is unclear how the instructions were presented or how the subjects were informed about the aim of the study.
3. Selection effects could also be a problem. However, it is not reported on what basis subjects were selected or even how subjects were recruited.
4. Translation effects could be a huge problem. Both questionnaires used the word "Qi" and are therefore likely to measure the same concept (at least to some degree).
5. The validity of data, exclusion criteria, and missing data could play a role but are not reported.
6. The main finding is the very high correlation between Qi and Flow experience but it is not clear how these scores were calculated from the sub scores.

**RESPONSE:** We agree with these suggestions and thank the reviewer for the constructive criticism that we did not make a clear description of the data collection procedures and statistical analysis in the manuscript. The following is our response and a revised point-by-point statement.

1. Sequencing effects: The first part of the questionnaire concerned inner feelings that described the participants’ present psychological state, comprising 36 flow questions and 14 Qi questions. The second part concerned basic information, such as gender, birth year, years practicing Qigong, and date at the time of practicing. The revised statements are in lines 285–289.
2. Expectation effects: The participants were asked to sign a Qi knowledge letter and an environmental experience informed consent form. They were then given the Qi-questionnaire package with 12 questionnaires that randomly assigned them to 12 urban green space experimental sites at NTU. The description in the consent form read as follows: We appreciate you joining this Qi and environment experience. This study is designed to understand your inner feelings about practicing Qigong. Our research data are only for academic use. Please pay attention to your physical condition and then practice breathing for 10 minutes at the randomly assigned experimental sites. You may practice breathing at 2-3 experimental sites per day from 7:00–11:00 in the morning, after which you may finish the questionnaire. For returning the completed Qi-questionnaire package, you will get a small gift. (lines 274–284)
3. Selection effects: In this study, participants were selected based on the criterion that they could sense the Qi state, even if only weakly. We assumed our participants could feel sense of Qi state stability in order to test the relationship between the Qi experience and the flow experience in multiple Qigong practicing. (lines 264–267)
4. Translation effects: In this study, the flow experience was measured as the challenge–skill balance that one achieves to gain an optimal experience state, which can be evoked by practicing Qigong. (lines 215–216). For the content validity of the revised FSS, we invited three Qigong novice practitioners to review the comprehensibility of each item, after which we invited three Qigong masters to determine the knowledge and skill level of the content tested in each question (lines 244–249) to reduce the translation effects. Moreover, we revised and used the words in the abbreviated descriptions of the items from the version in Tenenbaum, Fogarty, and Jackson (1999) to provide a clearer translation in the English version of practicing Qigong in the flow experience (Table 4).
5. The validity of data: Thank you for the suggestions. The description of the data collected was revised as follows: “We randomly assigned the 62 participants to 12 urban green space experimental sites at NTU. However, since four participants did not complete the experiment, a total 58 of participants (NTU students=40, Taipei Tan Tao Culture Research Association members=18, M=33.7 years old) were measured in the statistical analysis.” (lines 270–273). We also added “A total of 659 completed questionnaire packages were returned, for a return rate of 88.58%.” (lines 293–295) and “A total of 659 completed questionnaires packages (N=58, 11-12 times per person) were collected, including five incomplete responses.” (lines 298–299)
6. The high correlation between the Qi and flow experiences: We now state in the statistical analysis section and revise in more detail the sub-scores of the mean scores and standard deviations for all variables (see lines 317–336).

[6] Conclusion
The conclusion could be expanded to incorporate different explanations for the findings of the paper. In correlation designs, several types of causal (or not causal) links are possible. The structural relationship could be:
a) Flow is a part of the Qi-Gong experience (among others).
b) Flow and Qi are the same concept.
c) Qi-Gong evokes Flow depending on the skill of the practitioner.
d) Flow evokes Qi.
e) Physical activity (of any kind) leads to both Flow and Qi.
Probably several other connections are also plausible. These should be explored and discussed. Additionally, future research directions to address these explanations could prove interesting

**RESPONSE:** Thanks for the suggestions. The revised version of our correlation results is as follows: In the first paragraph, we summarize how we determined the results (lines 436–447) and explain our findings, “The Western concept of the flow experience in outdoor activities (Qigong) seems to be the same as that emphasized in East Asian disciplines, i.e., the Qi experience.” In the second paragraph, we explain the qualitative and quantitative methods that led to our findings (lines 448–456). The last paragraph gives suggestions for future research (lines 457–463).

[7] Grammar and formulations
While reading the paper I regularly stumbled upon punctuation mistakes or unfamiliar grammatical structures and sentences. Having studied the Chinese language myself, I know about the huge differences in grammar and wording compared to western languages. I would recommend the help of a native speaker to reduce linguistic mistakes. For example, line 62 “In Taoists” should be “In Taoist philosophy”; “In Taoism” or “For Taoists”.

**RESPONSE:** Thank you for the suggestions. The article will be submitted for ESL academic editing, which we believe will enhance the fluency and readability of the manuscript.

[8] line comments
45 “the mental state that influences overall health”: there is not just one mental state that can influence overall health.

**RESPONSE:** We revised line 45, “the mental state that influences overall health,” to “which are both part of the mental state that influences mental health, well-being, and self-consciousness.” (lines 60-61)

58 “sense of tao”: this concept should be explained for readers unfamiliar with Chinese philosophy.

**RESPONSE:** We restructured line 58, “sense of Tao,” and added it to lines 37; we then explained the concept of “sense of Tao,” which is “the laws of balance that harmonize rhythms in humans and nature.”

70 “represent special forms of the Flow experience”: Are there studies that analyzed this? If yes, you can go into detail since this seems very important for your research question. If no, on what is this claim based? And what does “a special form of the Flow experience” mean?

**RESPONSE:** We added line 70 to lines 114–119 to be more in line with your comments. We hope that the edited section clarifies the idea of “a special form of the flow experience.” According to the book, *Flow: The Psychology of Happiness* and what other activates (Tai Chi, Kung Fu, and Judo) could evoke the flow experience. Although we found that there was limited scientific literature on the analysis of Qigong and the flow, we did find a 2019 study that tested the emotional state and the flow experience during the practicing of Qigong. This study used open-ended questions to classify participants’ emotional state after practice of Qigong and also used PANAS and a flow scale to report participants’ feelings (see lines 119–125).

76 “the urban green environment”: is that some form of organization?

**RESPONSE:** We changed the misused word “supporter “to “representative” in line 252 and added a sentence to this paragraph, “The research selected 30 study sites to test the relationship between Qi and flow experience.”

126 “the words “Qi experience,” “Qi,” and “Qi practice” were added”: This could be a big problem. If you change the wording from “Flow” to “Qi” you probably don’t measure Flow anymore but Qi instead. Perhaps you should repeat the analysis without these items.

**RESPONSE:** Thank you for pointing out this problem that we can make clearly description in this point. The flow state scale was measured as challenge–skill balance that one achieves to attain the optimal experience state, which can be evoked by practicing Qigong (lines 215–216). Therefore, “These items were then translated into Chinese, with the items “Qi experience,” “Qigong practice,” and “Qi” added to describe the active status of the flow experience, which can be induced by practicing Qigong. The modified sentences, however, retained the main statement of the concept. For instance, “experience is extremely rewarding” is the main concept in autotelic experience; therefore, the question was modified as “I think practicing the Qi experience is extremely rewarding in this time.” (lines 235–237).

142 “were selected”: How did the selection process work? What were the selection criteria?

**RESPONSE:** Sorry for the confusing statements. The “selection process” is likely to be the “invited process.” The criterion for joining the study is that participants “could sense the Qi state, even if only weakly. “Sixty-two participants were recruited from a primary Qigong course at NTU and from the Taipei Tan Tao Culture Research Association.” (lines 264-268).

146 “letter of Qi and environmental experience consent”: Please explain what this consent includes

**RESPONSE:** The Qi letter and environmental experience consent form were revised according to lines 278–284: “We appreciate you joining this Qi and environment experience. This study is designed to understand your inner feelings about practicing Qigong. Our research data are only for academic use. Please pay attention to your physical condition and then practice breathing for 10 minutes at the randomly assigned experimental sites. You may practice breathing at 2-3 experimental sites per day from 7:00–11:00 in the morning, after which you may finish the questionnaire. For returning the completed Qi-questionnaire package, you will get a small gift.”

148 “from 7:00–11:00”: It does not seem necessary to go into detail about the theoretical foundation of the timeslot.

**RESPONSE:** For this point, we revised and added a separate paragraph to clarify our data collection procedures (lines 291–295), and we deleted the theoretical foundation for the time period used.

153 “After practicing, breathing for 10 minutes, finishing the questionnaires”: The description of the procedure is quite short and does not explain the procedure completely understandable. Please expand this point in a separate paragraph. Did the participants do Qigong for 4 hours or just at a certain time between 7 and 11 a.m.? What amount was the gift card and what for? In what order were the questionnaires answered? etc.

**RESPONSE:** Thanks for the suggestion. We separated this information into another paragraph. The duration of practicing Qigong, ordering of the questionnaires, and the amount of gift card were revised in lines 285-289.

161 “F=3.32”: What kind of analysis was used?

**RESPONSE:** Thank you for careful consider that we have chance to state the statistical analysis clearer. We used ANOVA to re-analyze the data. We revised our manuscript as follows: “The ANOVA on the number of years of Qigong practice indicated no significant influence on the flow experience: F(2, 651)=2.593, p=0.076, p>0.05, η²=0.008, for those practicing under half a year (N=24, n=276, M=3.466, SD=0.546), half a year to under five years (N=26, n=291, M=3.468, SD=0.604), and six years to over 15 years (N=8, n=87, M=3.622, SD=0.660). The ANOVA also revealed a group difference concerning the Qi experience: F(2, 651)=5.04, p=0.007, p<0.05, η²=0.015), for those practicing under half a year (N=24, n=276, M=3.553, SD=0.657), half a year to under five years (N=26, n=291, M=3.465, SD=0.724), and six years to over 15 years (N=8, n=87, M=3.737, SD=0.805). The Games-Howell post hoc test showed a significant difference between half a year to under five years and six years to over 15 years.” (lines 305–314).

188 “Furthermore, an outdoor environment with trees … induces a better Qi and Flow”: It is unclear where this insight comes from. Is it a quote, a result, or an intuition?

**RESPONSE:** Sorry for the unclear statement. The idea in this statement was moved to the “Limitations and Recommendations for Future Research” section in the “Discussion” (lines 412–422).

192 “Since urban green space plays a critical role in”: Urban green spaces are mentioned regularly but it is not clear why they are important to the research question. Qi as well as Flow can be experienced indoors.

**RESPONSE:** We revised our paper in lines71–76 (the reason for why we think urban green space is important to Qi), lines 143–146 (the importance of our aim), and lines 252– 256 (connecting the idea to our study sites) outlined by you and reviewer #3. We honestly feel that your insightful suggestions helped us in improving the manuscript to clarify the role that urban green space played in our study.

Table 3 “N=654”: I am confused about the sample size. You wrote about 58 subjects. Therefore, N should be 58.

**RESPONSE:** We revised N=58, thank you for the comment.

235 “used mixed methods”: It is unclear what mixed methods where used. The main body of the research seems to be based on questionnaires only.

**RESPONSE:** We clarified the statement in “Aim and Scope of the Study” (line 135) to illustrate how we measured the Qi experience: “We used the qualitative method to generate the Qi experience questionnaire and the quantitative method to test the correlation between the Qi experience and the flow experience” (lines 150–152).

Bibliography: Not in alphabetical order.

**RESPONSE:** Thank you for reminding us. We applied the correct formatting guidelines in the bibliography.

**Reviewer 3 Comments:**

Reviewer #3: The submitted study investigated the relationship between the eastern concept of Qi experience and the western concept of flow during Qigong. In a first step, they used qualitative methods to generate a questionnaire that assesses the Qi experience. In a second step, they assessed the Qi experience and the flow experience after Qigong practice in a group of students and Qigong masters in three different environments. They found high correlations between scales of the Qi experience questionnaire and the scales of the Flow State Scale, suggesting a large overlay of the concepts of Qi experience and flow during Qigong. The submitted study would enrich the present literature on Qi experience and flow. However, the manuscript would need some major revisions before being ready for publication. Especially, the manuscript would benefit from a clearer structure and more essential details in the methods and results section. This would also allow me to better evaluate the quality of the methods and analysis.
**RESPONSE:** We wish to thank you, Dr. Sonja Annerer-Walcher, for your constructive comments in this second round of review. Your comments provided valuable insights to help us refine the manuscript content and analysis. We revised the manuscript to provide more details and to give a clearer structure for readers.

1. I would welcome a clearer structure in the introduction and discussion.

**RESPONSE:** Thank you for the insight suggestion. We revised the structure, added headings, and added scientific research in the introduction to improve the statements in each paragraph. In lines 30–50, we address the concepts of the Qi and flow experiences and then summarize our research scope (lines 59–66). The paragraph beginning with line 67 addresses the concept of Qigong and the essence of the Qi experience; the paragraph starting with line 90 addresses the concept of the flow experience; the paragraph starting with line 113 addresses the comparison between the flow and Qi experiences and makes a clear statement from the literature review to the present research (lines 136–154). In the discussion, the first paragraph is a short summary of the research findings (lines 360–374) and is followed by two subheadings: “A Possible Explanation for the Results” (start from line 375) and “Limitations and Recommendations for Future Research.” (start from line 402)

2. As you have “urban green spaces” in your title, please introduce their role for the Qi experience in the introduction.

**RESPONSE:** We addressed the comments offered by you and reviewer #2. The urban green space plays an important role in the Qi experience, as written in lines 71–76. The aim is restructured in lines 143–148, and we connect the idea in our study sites (lines 252–256). We are grateful for your comments.

3. Please add details and structure to the methods section.

**RESPONSE:** The reviewer made a good suggestion. We apologize for the lack of details and structure in the “Methods” section. In the revised version, we expanded descriptions in *“*Developing Qi Experience Questionnaires” with the definition of the Qi experience (lines 157-158), how many open-ended questions participants responded to (lines 159–164), and a revision to Table 1, separating it into two tables (1 and 2) with clearer Qi experience descriptions. Besides, we used MTMM to assess construct validity to strengthen the Qi experience questionnaires (lines 187–207 and Table 3). In the “Instruments of the Flow State Scale (FSS),” we added the definition of the flow experience in the first sentence (lines 215-216) and, in the second paragraph, we added the statement of content validity (lines 244–249). We also added a paragraph for our data collection (lines 291–295). In the “Participants” section, we added a more detailed description of subjects—for instance, how many subjects participants in open-end data collecting and rejoin the study in this time (lines 268-270), missing data (line 271), a brief description of the consent form (lines 278–284), and the sequence of the questionnaires (lines 285–288).

4.For example, it is not clear how many students were asked the open questions for the generation of the Qi experience questionnaire. Were there two groups of 26 students each, so 52 in total? Were they asked once only, once after practice in each of the three environments, or even multiple times in each environment?

**RESPONSE:** We are grateful for your comments and have revised our paper accordingly. We revised the paragraph: “The study asked the sampled students to, in one sentence or adjective, describe their Qi experience by answering the open-ended questions on the Qi experience questionnaire. Data were collected on 12 occasions during the late spring and late fall (at the end of the semester) from students we considered to be more familiar with the Qi experience. Each primary Qigong course included 26 students who, after practicing, were asked to respond to the open-ended questions on the questionnaire twice in three different practice environments at NTU: (a) indoors, (b) a built environment within natural surroundings, and (c) the natural environment, beside an ecological pond.” (see lines 159–166). However, should we present more detailed information to describe the participants? For example: “Each primary Qigong course included 26 students; however, students were absent sometimes. The range of attending the class and answering the open-ended questions was 16-26 participants per time per practice sites.” Thank you.

5. Participants in the questionnaire study: Please add some details to this section. Did participants participate for only one session or multiple times? Were they always assigned to the same site or did the site change for participants? How often did they participate on average? Were students and masters evenly distributed across sites? Were the students the same as in the open-ended questions study?

**RESPONSE:** Thanks for the suggestion about adding more details in this study. First, we apologize for the inappropriate classification of “students and masters” in the last version. We decided re-catelog “students” or “masters” because there are students who have practiced for years. Therefore, we used the organization instead. To recheck the data, 40 students and 18 participants form Taipei Tan Tao Culture Research Association joined our research. In this study, we randomly assigned participants to 12 urban green space experimental sites at NTU (lines 270–271). In the brief description on the consent form, participants were asked to practice breathing at 2-3 experimental sites per day from 7:00–11:00 in the morning and then finish the questionnaires. The revised, detailed descriptions are on lines 278–284. “Thirteen of the 52 students who participated in the study on perceptions of the Qi experience were willing to participate in this study.” (lines 268–270)

6. Results: “A total of 654 valid data were collected (n=654 per person-time)”. Does this mean there were 654 completed questionnaires packages overall?

**RESPONSE:** This is a good point, one which led us to rewrite this sentence. We apologize for not being clearer. The revised sentence is as follows: “A total of 659 completed questionnaires packages (N=58, 11-12 times per person) were collected, including five incomplete responses. Therefore, 654 valid packages (n=444 (67.9%) males; n=210 (32.1%) females) were measured in the statistical analysis.” (lines 298–300).

7. In the last sentence of the results section you mention that the outdoor environment had a positive effect. Please add data and results of this analysis.

**RESPONSE:** Thanks for the suggestion. The idea should be present in the “Discussion” section; therefore, we revised it (lines 412–422) in the second paragraph in “Limitations and Recommendation for Future Research.”

8. Although you state that all data is available, I could not find any reference that would allow me to access the data.

**RESPONSE:** In line with the suggestion by reviewer #2, we now give details of the results in “A Descriptive Picture of the Data” (start from line 297) and “High Correlation Between the Flow and Qi Experiences” (start from line 316) and in Table 5. We hope all the relevant data within the manuscript are clearer for readers. Please see S1 Appendix for the row data information.

**Reviewer 4 Comments:**

Reviewer #4: Qi experience is an interesting topic. The study analyzed the relationship between the Qi experience and the flow experience by using quantitative and qualitative methods. However, the result of correlation between the flow and Qi experiences is insufficient to support the final conclusion, especially for the placebo effect.

**RESPONSE:** We would like to thank reviewer #4 for the effort and time he/she put into helping us to revise our manuscript and especially to give more details about how we measured the Qi experience and the flow experience. A good point was also raised about the placebo effect. For this point, we added some statements in the discussion (lines 403–411).

Method:
1. Data were collected 12 times within the last spring and late fall, and in different practice environments, including (a)indoors, (b) a built environment with nature, and (c) the natural environment at NTU (line 85-87). According to the authors’ description, Qigong is a type of mind-body exercise that emphasizes a harmonious interaction between humans and the environment (line 54-55).
Are there any differences of the data among different environment or among different time points?

**RESPONSE:** This is a good point, one which led us to rewrite this section of the paper. As we mention a harmonious interaction between humans and the environment and the effect of the Qi experience, we revised the statement in Table 2 as follows: “The research time we chose was late fall in the indoor environment (Fig 1) and in the outdoor environment (Fig 2) about 25.7°C and 20.8°C; outdoor environment (Fig 3) is about 23.8°C and 26.1°C; in late spring outdoor environment (Fig 2) is about 30.8°C and 33.3°C; outdoor environment (Fig 3) is about 29.4°C and 25.1°C. The data were measured by the Central Weather Bureau. When asked for one sentence or adjective to describe their Qi experience, subjects mentioned that by practicing in outdoor environments, especially within nature, they can feel a stronger Qi experience than indoors. However, a few subjects thought the outdoors was a little too hot or cold.”

2. The Flow State Scale was translated into Chinese (line 126). Has the Chinese version been revised and tested for reliability and validity?

**RESPONSE:** Thank you for the suggestion. We added the following sentence to the paragraph on reliability and validity: “For the content validity of the revised FSS, we invited three Qigong novice practitioners to review the comprehensibility of each item, after which we invited three Qigong masters to determine the knowledge and skill level of the content tested in each question. We recruited 58 subjects within 654 valid data to test the revised FSS, which showed very good reliability (Cronbach’s alpha=0.976).” (lines 244–249)

Results:
3. Please check which p value is correct: For the number of years practicing Qigong, the results show significant influence on the flow experience (F=3.32* p<0.05, p <0.01, η2=0.010) (line 161).

**RESPONSE:** Thank you for the comments. We apologize for the unclear information. We added the following sentence to the paragraph on statistical analysis that reviewer #2 and reviewer #3 addressed: “The ANOVA on the number of years of Qigong practice indicated no significant influence on the flow experience: F(2, 651)=2.593, p=0.076, p>0.05, η²=0.008, for those practicing under half a year (N=24, n=276, M=3.466, SD=0.546), half a year to under five years (N=26, n=291, M=3.468, SD=0.604), and six years to over 15 years (N=8, n=87, M=3.622, SD=0.660). The ANOVA also revealed a group difference concerning the Qi experience: F(2, 651)=5.04, p=0.007, p<0.05, η²=0.015), for those practicing under half a year (N=24, n=276, M=3.553, SD=0.657), half a year to under five years (N=26, n=291, M=3.465, SD=0.724), and six years to over 15 years (N=8, n=87, M=3.737, SD=0.805). The Games-Howell post hoc test showed a significant difference between half a year to under five years and six years to over 15 years.” (lines 305–314)

4. The p values should not be 0.0. (line 162). Maybe you can use p<0.001 instead.

**RESPONSE:** Thank you for the comments. We apologize for the confusing information. We have revised line 306 and line 310.

5. There is an important problem that all the subjects have received Qigong training, and the description of Qi is also included in The Flow State Scale. Without the control group, the subjects may know the purpose of the study, and be affected by the placebo effect.

**RESPONSE:** Thank you for your concern. We added more details to address how we invited the participants and the criteria that the participants should meet (See lines 264–267). Although our correlation of the Qi experience and the flow experience was high, the effect could have been a placebo effect. “The criteria that were used to recruit participants attempt to avoid the placebo effect. Moreover, the results led us believe there were difference between novice (practicing under half a year) and master (practicing more than six years) practitioners in their experience of the sense of Qi, indicating that the assessment process was reliable.” (lines 407–411)
